# Activation of the “Splenocardiac Axis” by electronic and tobacco cigarettes in otherwise healthy young adults

**DOI:** 10.14814/phy2.13393

**Published:** 2017-09-12

**Authors:** Zachary Boas, Pawan Gupta, Roya S. Moheimani, May Bhetraratana, Fen Yin, Kacey M. Peters, Jeffrey Gornbein, Jesus A. Araujo, Johannes Czernin, Holly R. Middlekauff

**Affiliations:** ^1^ Division of Cardiology Department of Medicine David Geffen School of Medicine at UCLA Los Angeles California; ^2^ Department of Molecular and Medical Pharmacology Ahmanson Translational Imaging Division David Geffen School of Medicine at UCLA Los Angeles California; ^3^ David Geffen School of Medicine at UCLA Los Angeles California; ^4^ Department of Environmental Health Sciences School of Public Health Los Angeles California; ^5^ Department of Biomathematics David Geffen School of Medicine at UCLA Los Angeles California

**Keywords:** Arterial inflammation, electronic cigarettes, FDG‐PET/CT, hematopoietic activation, tobacco cigarettes

## Abstract

The “Splenocardiac Axis” describes an inflammatory signaling network underlying acute cardiac ischemia, characterized by sympathetic nerve stimulation of hematopoietic tissues, such as the bone marrow and spleen, which then release proinflammatory monocytes that populate atherosclerotic plaques, thereby promoting ischemic heart disease. Electronic (e) cigarettes, like tobacco cigarettes trigger sympathetic nerve activation, but virtually nothing is known about their influence on hematopoietic and vascular tissues and cardiovascular risks. The objective of this study was to determine if the Splenocardiac Axis is activated in young adults who habitually use either tobacco or e‐cigarettes. In otherwise healthy humans who habitually use tobacco cigarettes or e‐cigarettes (not both), we used ^18^F‐flurorodeoxyglucose positron emission tomography/computer tomography (FDG‐PET/CT) to test the hypothesis that tobacco or e‐cigarettes increased metabolic activity of the hematopoietic and vascular tissues. FDG uptake in the spleen increased from nonuser controls (1.62 ± 0.07), to the e‐cigarette users (1.73 ± 0.04), and was highest in tobacco cigarette smokers (1.82 ± 0.09; monotone *P* = 0.05). Similarly, FDG uptake in the aorta increased from the nonuser controls (1.87 ± 0.07) to the e‐cigarette users (1.98 ± 0.07), and was highest in tobacco cigarette smokers (2.10 ± 0.07; monotone *P* = 0.04). FDG uptake in the skeletal muscle, which served as a control tissue, was not different between the groups. In conclusion, these findings are consistent with activation of the Splenocardiac Axis by emissions from tobacco cigarettes and e‐cigarettes. This activation suggests a mechanism by which tobacco cigarettes, and potentially e‐cigarettes, may lead to increased risk of future cardiovascular events.

## Introduction

**Figure 1:**
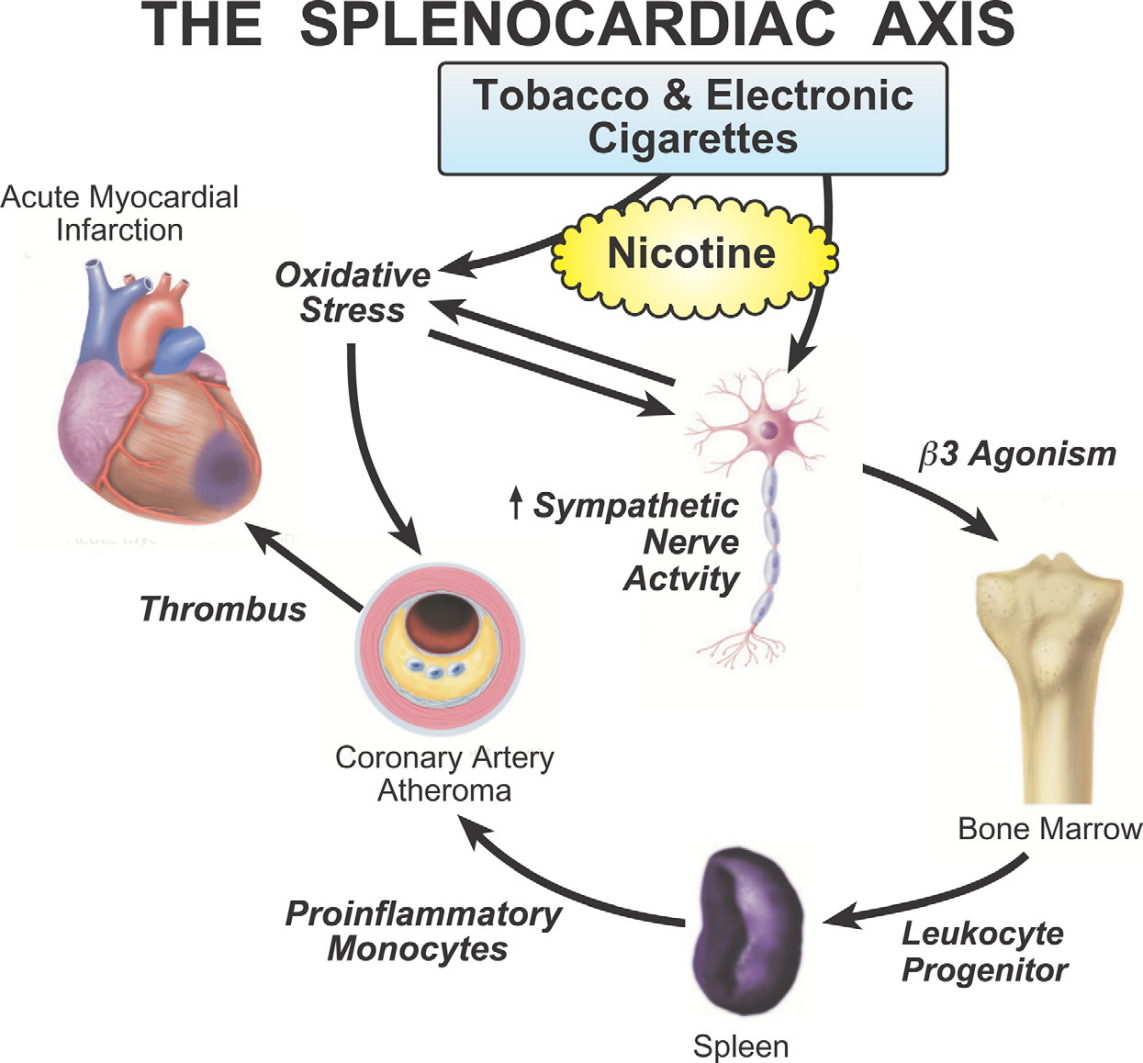
Splenocardiac Axis. The Splenocardiac Axis is an inflammatory signaling network, initiated by increased sympathetic nerve activity (SNA), that underlies the development of atherosclerosis and acute myocardial ischemia. Nicotine, from tobacco and electronic cigarettes, increases SNA directly, and through activation of oxidative stress. Increased SNA mobilizes bone marrow progenitor cells, which migrate to the spleen, where they multiply. Proinflammatory monocytes enter the circulation, reaching the arterial wall, where increased monocytes, oxidative stress, and prothrmombotic factors, promote atherosclrosis. (Figure adapted, with permission, from reference Libby et al. 2016).

Using an integrative biological approach to inflammation, the existence of a signaling network, termed the “Splenocardiac Axis,” linking the brain, autonomic nervous system, bone marrow, and spleen to the development of atherosclerosis and acute myocardial infarction, has been proposed (Fig. 1) (Libby et al. 2016). Recent studies in rodent models support the concept that during acute stress, increased central sympathetic outflow activates bone marrow progenitor cells and leukocytes via *β*‐3 receptor stimulation (Dutta et al. 2012; Laukova et al. 2012). The leukocyte progenitor cells migrate from the bone marrow to the spleen, where they multiply in response to stem cell factors. Augmented numbers of proinflammatory monocytes then leave the spleen to enter the circulation, reaching the arterial wall where increased monocyte recruitment promotes and accelerates atherosclerosis. Not simply a transient process, the proinflammatory changes detected in the blood vessel wall have been found to persist for months (Dutta et al. 2012; Laukova et al. 2012; Heidt et al. 2014; Libby et al. 2016).

This Splenocardiac Axis may explain the observation in humans that heightened sympathetic tone, for example, that accompanies acute or chronic mental stress, pain, or even an acute myocardial infarction, is a risk factor for future acute ischemic cardiovascular events (Rosengren et al. 2004; Grassi et al. 2015) (Thune et al. 2011). Emami and colleagues used ^18^F‐flurorodeoxyglucose positron emission tomography/computer tomography (FDG‐PET/CT) to demonstrate augmented inflammatory activity in the spleen and arterial wall in patients following an acute coronary syndrome compared to control subjects (Emami et al. 2015). Furthermore, increased splenic metabolic activity in patients was an independent predictor of adverse cardiovascular events during follow up (Emami et al. 2015).

Tobacco cigarettes, the most common preventable risk factor for premature cardiovascular death in the United States, produce a relative hyperadrenergic state (Middlekauff et al. 2014). Nicotine, one of the 7000 constituents present in tobacco cigarette smoke, acts on receptors in the brain, autonomic ganglia, and sympathetic nerve terminals to increase adrenergic tone and norepinephrine release (Haass and Kubler 1997; Middlekauff et al. 2014). Furthermore, previous reports have confirmed that tobacco cigarette smoking is associated with a leukocytosis (Higuchi et al. 2016). We reasoned that in habitual tobacco cigarette smokers who are regularly exposed to nicotine, activation of the Splenocardiac Axis and arterial inflammation may be present and is detectable and quantifiable by FDG‐PET/CT, explaining in part the increased risk for acute coronary syndromes and sudden death conferred by smoking.

Importantly, a new tobacco product, the electronic (e) cigarette, is gaining skyrocketing popularity, especially among young people, but the cardiovascular risk associated with e‐cigarettes remains unknown. Although e‐cigarettes deliver much lower levels of toxicants including carcinogens, compared to tobacco cigarettes (Goniewicz et al. 2014), they typically deliver nicotine. Recent evidence from our laboratory supports the concept that habitual e‐cigarette users who do not smoke tobacco cigarettes also have increased sympathetic activation (Moheimani et al. 2017). Accordingly, we hypothesized that in habitual e‐cigarette users, who do not smoke tobacco cigarettes, increased hematopoietic and vascular metabolic activity may be intermediate between tobacco cigarettes smokers and nonsmokers, identifying a mechanism by which e‐cigarettes may too increase future cardiovascular risk.

## Materials and Methods

### Study population

Otherwise healthy habitual tobacco cigarette smokers or habitual e‐cigarette users (not dual users) between the age 21 and 45 years, who had used tobacco cigarettes or e‐cigarettes, respectively, most days for a minimum of 1 year, in whom plasma cotinine levels were elevated, were eligible for the study if they met the study criteria: (1) no known health problems, (2) nonobese (≤30 kg/m^2^ BMI), (3) not taking prescription medications except oral contraceptive pills, (4) alcoholic intake ≤2 drinks per day and no illicit drug use, and (5) not exposed to secondhand smoke, or using licensed nicotine replacement therapies. Healthy volunteers meeting these inclusion criteria, who were not e‐cigarette users or tobacco cigarettes smokers, were eligible to be enrolled as nonuser controls. Participants who were former tobacco cigarette smokers were eligible for the study if they had quit smoking >1 year prior to the study. Participants were enrolled with the goal to balance age and sex among the groups. The experimental protocol was approved by the Institutional Review Board at the University of California, Los Angeles and written informed consent was obtained from each participant. The study is registered at ClinicalTrials.gov (NCT02734888).

### FDG‐PET/CT imaging

FDG‐PET/CT imaging was performed according to previously reported standards and guidelines to optimize FDG uptake in the hematopoietic tissues and arterial wall (Tawakol et al. 2013; Bucerius et al. 2014; Emami and Tawakol 2014; Tarkin et al. 2014; Chen and Dilsizian 2015; Emami et al. 2015; Huet et al. 2015). Briefly, following an overnight fast, and confirmation of fasting blood glucose <200 mg/dL, 0.14 mCi/kg of FDG was injected intravenously. The subject rested without unnecessary motion for 90 min and then images of the neck, chest, and abdomen were obtained. High count 5‐min scans per bed position were obtained, compared to shorter acquisition of 2 min, or less, typically done for oncology imaging, producing higher count rate, and decreased image noise, resulting in better image quality for more reliable and reproducible quantitative assessment.

### Image analysis

All scans were read by a single investigator (P.G.) blinded to participant identification or study group affiliation. As previously reported, inflammation of the aorta, spleen, vertebral bone marrow, and adjacent erector spinae skeletal muscle (control tissue) were measured by placing a region of interest over axial sections (Tawakol et al. 2013; Bucerius et al. 2014; Emami and Tawakol 2014; Emami et al. 2015). For the aorta, measurements were made every 5 mm, starting 1 cm above the aortic valve annulus, continuing to the bottom of the aortic arch. The maximum standardized uptake value (SUV_max_) was recorded for each region of interest (Chen and Dilsizian 2015; Huet et al. 2015). Parenthetically, the sample size calculations (below) were based on the previously reported (Tawakol et al. 2013) variable “most diseased segment–tissue to background ratio (MDS‐TBR).” The TBR was calculated as a time‐ and dose‐corrected tissue radioactivity ratio of the SUV_max_ of the arterial wall compared to background superior vena cava venous SUV mean activity, and MDS was the 1.5 cm segment with the highest TBR and each 1.5 cm segment of either side. Since the time that these sample size calculations were performed, it has been argued that the SUV_max_ of the aorta is the preferred method of analysis (Chen and Dilsizian 2015; Huet et al. 2015). Both analyses are reported, herein. For the spleen, bone marrow, and skeletal muscle control, the SUV_max_ in the axial plane was measured. Analysis of FDG in the carotid arteries was neither planned nor attempted since identification and measurement of the carotid vessel wall metabolic activity, in the absence of intravenous contrast administration, is poor and prone to error, such as partial volume artifact.

### Blood tests

Venipuncture was performed by trained Nuclear Medicine staff on the day of the study. Blood was tested for glucose, and sent to the UCLA Clinical Laboratory for measurement of (1) cotinine (*t*_1/2_ 20 h), (2) carboxyhemoglobin (COHb, marker for tobacco cigarette, but not e‐cigarette use), (3) inflammatory markers, including C‐reactive protein (CRP) and fibrinogen. Blood samples were also centrifuged to separate into plasma samples, which were frozen at −80°C in a cryopreservative solution (Breton et al. 2014) for later analysis for the following antioxidant parameters: (1) paraoxonase‐1 activity, (PON‐1 activity), a protective ester hydrolase enzyme associated with HDL in blood that prevents the formation of oxidized LDL (Watson et al. 1995) (described below) (2) LDL Oxidizability (LDL‐Ox), indicative of susceptibility of apoB‐containing lipoproteins to oxidation as previously reported(Yin et al. 2013), (3) HDL antioxidant/anti‐inflammatory capacity, expressed as a HDL antioxidant index (HOI), which assesses the ability of HDL to inhibit LDL oxidation.

### Paraoxonase‐1 (PON‐1) enzymatic activity

The enzymatic activity of human plasma PON‐1 was determined by its capacity to hydrolyze paraoxon substrate to *p*‐nitrophenol. Assays were performed in duplicate in clear, flat‐bottom, 96‐well plates (Corning^®^ Costar^®^), and measurements were conducted using the BioTek Synergy Mx microplate reader and Gen5 software. From each plasma sample, 5 *μ*L was incubated with paraoxon (Chem Service Inc., catalog # N‐12816‐100MG) in the assay buffer (0.1 mol/L Tris‐HCl buffer at pH 8.5, with 2 mol/L NaCl and 2 mmol/L CaCl_2_) at room temperature. The kinetics of *p*‐nitrophenol formation were immediately measured every 15 sec at 405 nm for a total of four minutes in the BioTek microplate reader. The absorbance readings (OD/min) were converted into nanomoles *p*‐nitrophenol/(min·mL) with the use of the molar extinction coefficient for *p*‐nitrophenol, determined to be 16,734 mol/L‐1 cm‐1 at a pH of 9.18, and a path length of 0.58 cm.

### Sample size calculation

In a retrospective analysis of FDG‐PET/CT scans performed for clinical purposes, aortic inflammation was estimated by MDS‐TBR (Tawakol et al. 2013). In tobacco cigarette smokers (*n* = 8), MDS‐TBR was 1.34 with SD of 0.16, and nonsmokers (*n* = 9), MDS‐TBR was 1.17 with a SD of 0.12. Thus, we computed that a sample size of eight per group allowed confirmation a difference of 22% and 10 per group allowed confirmation of a difference of 8% between groups, assuming 80% power using a two‐sided alpha = 0.05. Our analysis included nine per group.

### Statistical analysis

Means were compared across controls, e‐cigarette users, and tobacco users using an analysis of variance model with ordered trend *F* tests. Under this model, *F* tests for ordered, monotone dose trend (control, e‐cigarette users, and tobacco cigarette smokers) were computed where the null hypothesis of no change was tested against the alternative of monotone change. The monotone test was used since the response should be ordered, where the group having the intermediate exposure or level should also have the intermediate response, at least on average. The mean and its standard error (SEM) are reported. For comparing binary data such as gender, *P* values were computed using Fisher's exact test. Associations of continuous variables with cotinine were assessed using the Spearman (*r*_s_) correlation. Differences or associations were considered statistically significant when *P* ≤ 0.05.

## Results

### Study population (Fig. 1)

**Figure 2:**
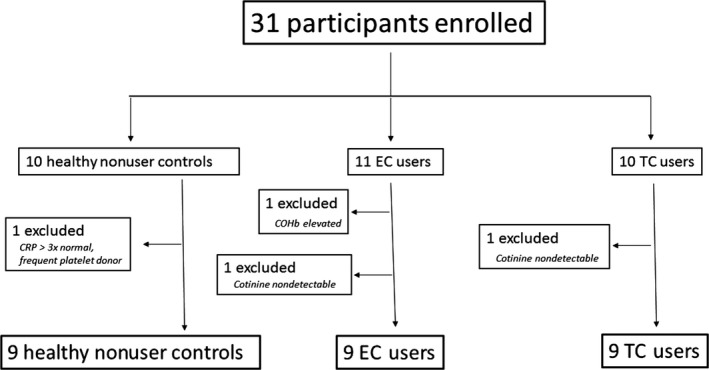
Patient enrollment. Thirty‐one participants were enrolled in the study. One was excluded from the healthy nonuser control group when her CRP returned threefold normal values, and it was learned that she was a frequent platelet donor. Two participants were excluded from the e‐cigarette group: one had elevated plasma COHb consistent with tobacco cigarette smoking (SRNT Subcommittee on Biochemical Verification, 2002), and one without detectable plasma cotinine level, indicative of insufficient e‐cigarette exposure. One participant was excluded from the tobacco cigarette group due to undetectable plasma cotinine level, indicative of insufficient tobacco cigarette exposure. COHb, carboxyhemoglobin; CRP, C‐reactive protein; EC, electronic cigarette; TC, tobacco cigarette.

A total of 31 participants meeting the above criteria were initially enrolled in this study, including 10 habitual tobacco cigarette users, 11 habitual e‐cigarette users, and 10 healthy control subjects. Nine in each group were included in the final analysis (see Fig. 2).

### Baseline characteristics (Table 1)

**Table 1:** Study population characteristics

	Nonuser Control (*n* = 9)	e‐cigarette user (*n* = 9)	T‐cigarette smoker (*n* = 9)	*P* value
Age (years)	28 ± 1.6	29 ± 1.5	27.1 ± 1.6	0.80
Sex (M/F)	6/3	7/2	7/2	0.82
Ethnicity				
African American	0	1	0	
Asian	2	1	1	
Hispanic	1	1	1	
White (non‐Hispanic)	6	6	7	
Cotinine (ng/mL)	0	120.4 ± 31.6	192.0 ± 55.8	0.18
Present e‐cigarette use				
Minutes/day	85 ± 30 (20–300)	NA	NA	
Duration (years)	2.1 ± 1.0 (1–4)	NA	NA	
Present T‐cigarette use				
Pack‐Years	NA	NA	7.3 ± 3.2 (0.7–30)	
SBP (mmHg)	112.7 ± 4.5	112.6 ± 4.4	120.3 ± 5.8	0.47
DBP (mmHg)	70.4 ± 2.9	71.3 ± 3.0	73.6 ± 3.9	0.77
MAP (mmHg)	84.4 ± 3.3	84.9 ± 3.3	87.7 ± 4.3	0.85
HR (bpm)	66.5 ± 4.2	61.1 ± 2.6	64.5 ± 3.4	0.55
Glucose (mg/dL)	85.9 ± 1.9	87.8 ± 2.9	88.7 ± 3.0	0.75
Fibrinogen (mg/dL)	239.7 ± 16.3	262.8 ± 10.7	247.6 ± 11.9	0.67
LDL‐Ox (units)	2725 ± 334	2365 ± 131	2801 ± 370	0.91
HOI (units)	1.36 ± 0.17	0.99 ± 0.25	1.24 ± 0.17	0.62
CRP (mg/dL)	0.31 ± 0.01	0.30 ± 0.00	0.41 ± 0.10	0.32

bpm, beats per minute; CRP, C‐reactive protein; DBP, diastolic blood pressure; HOI, HDL antioxidant index; HR, heart rate; LDL‐Ox, LDL oxidizability; MAP, mean arterial pressure; SBP, systolic blood pressure; T‐Cigarette, tobacco cigarette.

The nonuser control, habitual e‐cigarette user, and tobacco cigarette smoker groups were intentionally well‐matched by age and sex. Cotinine levels, an estimate of nicotine burden, were not different between the tobacco cigarette and e‐cigarette groups.

### Hematopoietic tissue metabolic activity is increased in tobacco and e‐cigarette users (Figs. 3 and 4)

**Figure 3:**
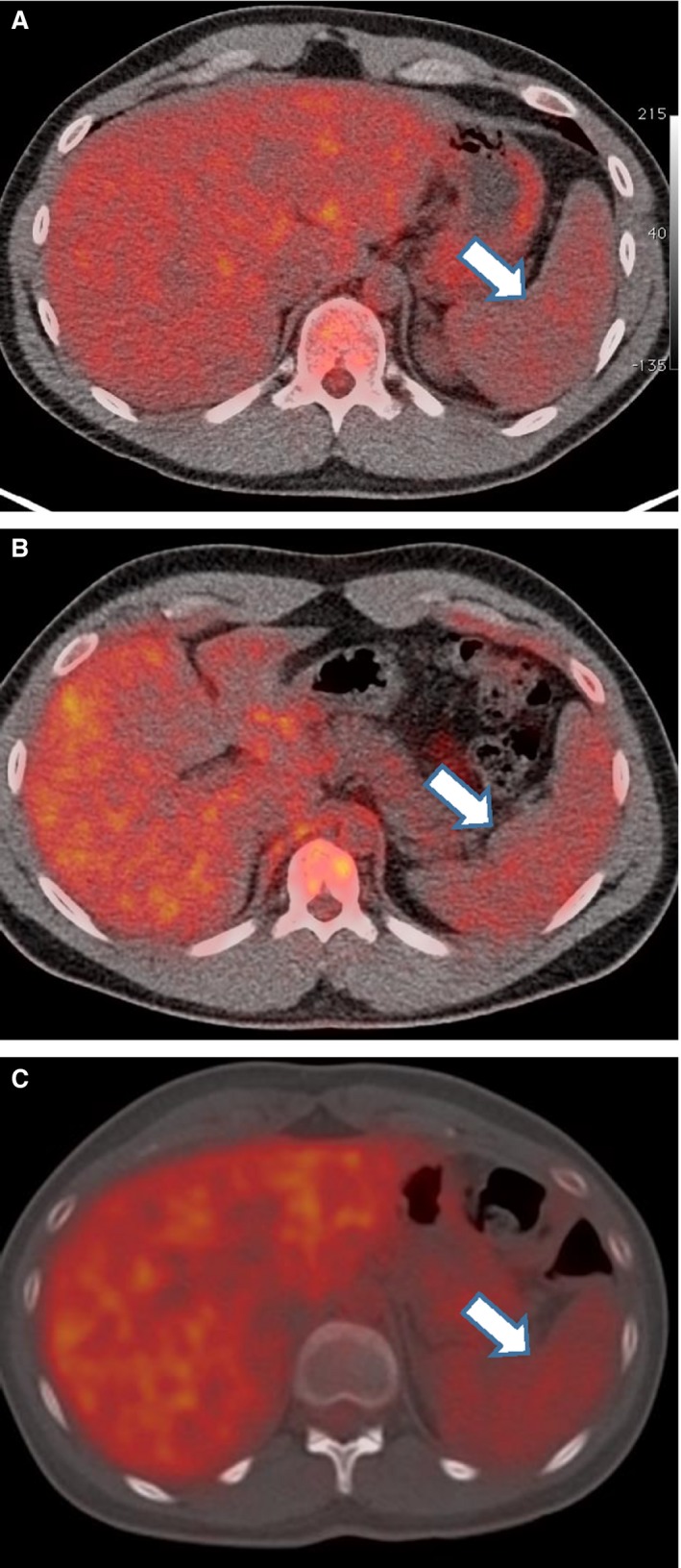
Panels A–C. Representative cross‐sectional PET scans from a nonuser (Panel A), e‐cigarette user (Panel B) and tobacco cigarette smoker (Panel C). The white arrow in each panel identifies the spleen.

**Figure 4:**
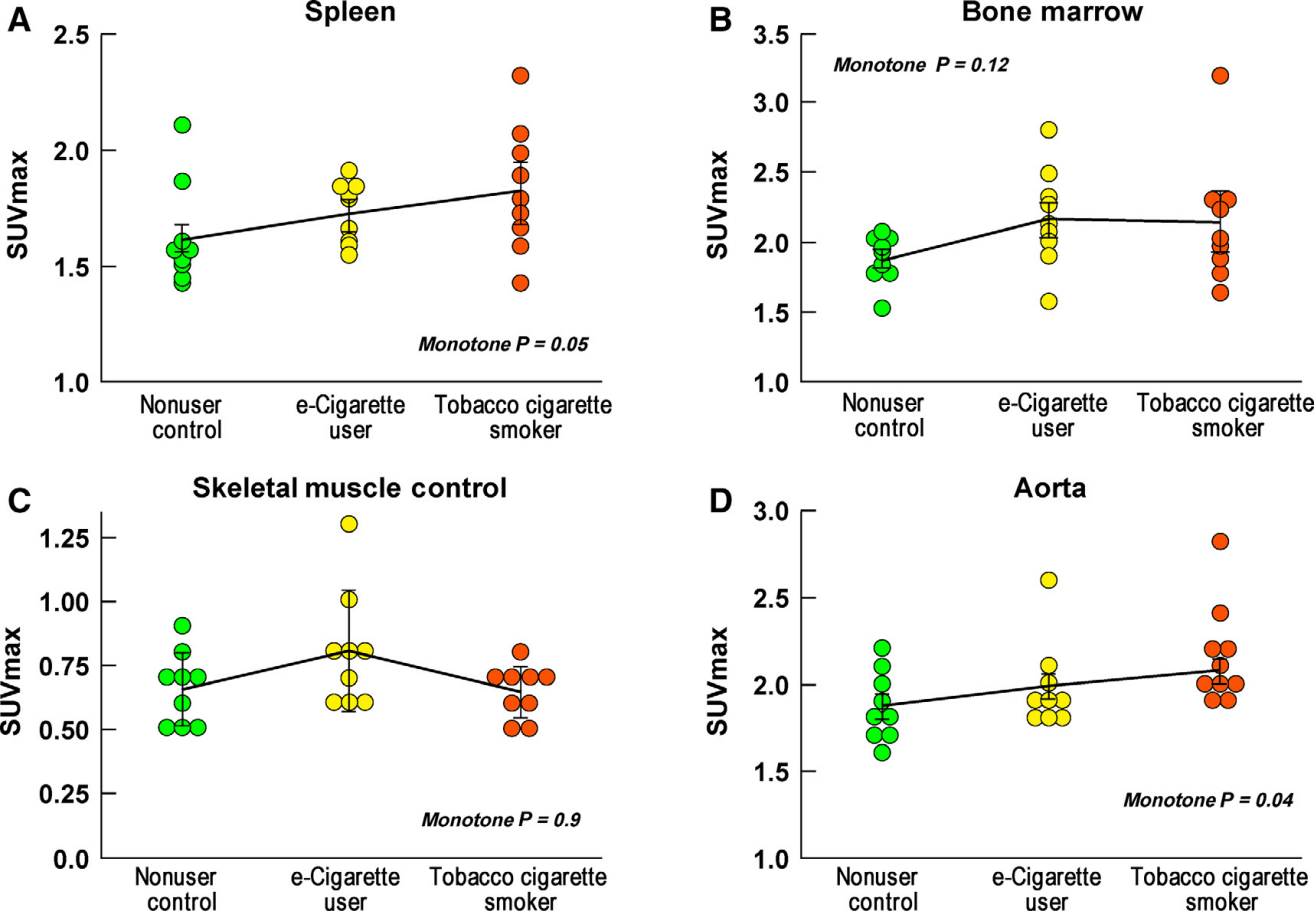
Activation of the Splenocardiac Axis in e‐cigarette and tobacco cigarette Users. Panel 4A. FDG uptake in the spleen increased from nonuser controls (1.62 ± 0.07), to the e‐cigarette users (1.73 ± 0.04), and was highest in tobacco cigarette smokers (1.82 ± 0.09; monotone *P* = 0.05). The individual between group comparisons were: tobacco cigarette smokers versus the nonuser controls, *P* = 0.056, e‐cigarette users and controls, *P* = 0.29, e‐cigarette users and tobacco cigarette smokers, *P* = 0.35. Panel 4B. FDG uptake in the bone marrow was lowest in the controls (1.88 ± 0.06) and was higher in both the e‐cigarette users (2.17 ± 0.12) and the tobacco cigarette smokers (2.14 ± 0.15), but the monotone *P* did not reach statistical significance (*P* = 0.12). The individual between group comparisons were: e‐cigarette users versus nonuser controls, *P* = 0.09, tobacco cigarette smokers versus the nonuser controls, *P* = 0.12, e‐cigarette users versus nonuser controls, *P* = 0.85. Panel 4C. As expected, FDG uptake in skeletal muscle, which served as a control tissue, was not different between the groups. Panel 4D. FDG uptake in the aorta increased from nonuser controls (1.87 ± 0.07) to the e‐cigarette users (1.98 ± 0.07), and was highest in tobacco cigarette smokers (2.10 ± 0.07; monotone *P* = 0.04). The individual between group comparisons were: tobacco cigarette smokers versus the nonuser controls, *P* = 0.04, e‐cigarette users and controls, *P* = 0.27, e‐cigarette users and tobacco cigarette smokers, *P* = 0.32. FDG = ^18^F‐flurorodeoxyglucose, SUV_max_ = maximum standardized uptake value

Representative cross‐sectional PET images from a nonuser, e‐cigarette user, and tobacco cigarette smoker are displayed in Figure 3. FDG uptake in the spleen as measured by SUV_max_ increased from nonuser controls (1.62 ± 0.07) to the e‐cigarette users (1.73 ± 0.04), and was highest in tobacco cigarette smokers (1.82 ± 0.09; monotone *P* = 0.05; Fig. 4A). FDG uptake in the bone marrow as measured by SUV_max_ was lowest in the controls (1.88 ± 0.06) and was higher in both the e‐cigarette users (2.17 ± 0.12) and the tobacco cigarette smokers (2.14 ± 0.15), but the monotone trend did not reach statistical significance (*P* = 0.12; Fig. 4B). FDG uptake as measured by SUV_max_ in skeletal muscle, which served as a control tissue, was not different between the groups (Fig. 4C).

### Aortic wall metabolic activity is increased in tobacco and e‐cigarette users

Aortic wall metabolic activity as measured by SUV_max_, was increased from nonuser controls (1.87 ± 0.07) to the e‐cigarette users (1.98 ± 0.07), and was highest in tobacco cigarette smokers (2.10 ± 0.07; monotone *P* = 0.04; Fig. 4D). When measured by MDS‐TBR, aortic wall metabolic activity was not different among the three groups (nonuser controls (1.87 ± 0.05, e‐cigarette users (1.81 ± 0.09), tobacco cigarette smokers (1.91 ± 0.09; monotone *P* = 0.75).

### Relationship of hematopoietic tissue metabolic activity with cigarette burden

Plasma cotinine, an estimate of tobacco cigarette and e‐cigarette burden, was weakly correlated with bone marrow activity (*r*_s _= 0.39, *P* = 0.05), but other correlations were not significant (data not shown).

### Markers of inflammation and oxidative stress (Table 1, Fig. 4)

**Figure 5:**
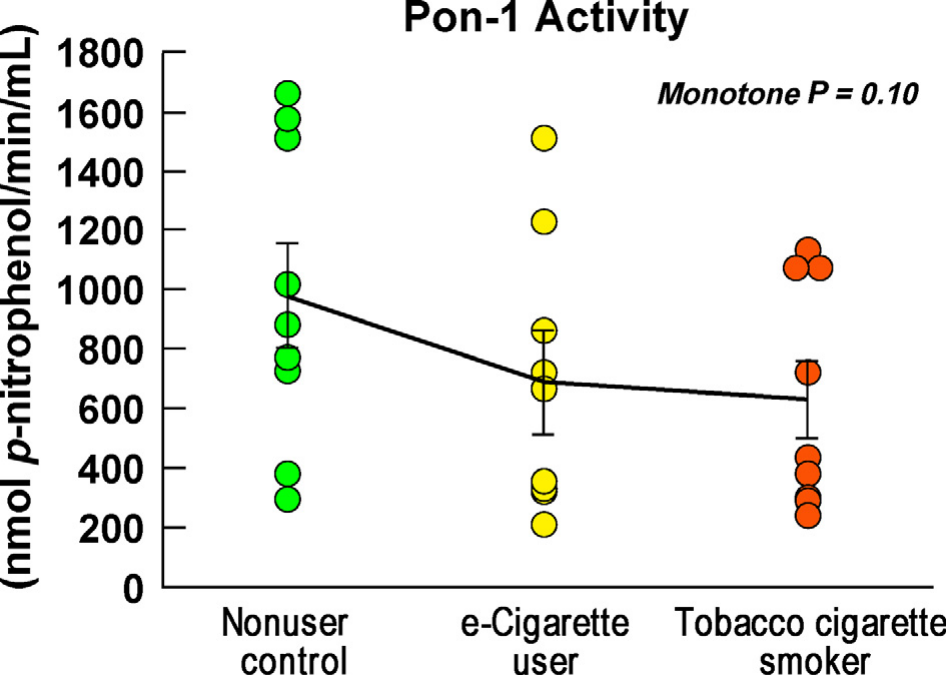
Oxidative stress. PON‐1 activity, a protective antioxidant enzyme, tended to be higher in the nonuser control group (971.6 ± 169 nmol *p*‐nitrophenol/(min·mL), intermediate in the e‐cigarette users (682.6 ± 169 nmol *p*‐nitrophenol/(min·mL), and lowest in the tobacco cigarette users (618.0 ± 125.7 nmol *p*‐nitrophenol/min·mL), monotone *P* = 0.10). The individual between group comparisons were: e‐cigarette users versus nonuser controls, *P* = 0.16, tobacco cigarette smokers versus the nonuser controls, *P* = 0.17, tobacco cigarette smokers versus e‐cigarette users, *P* = 0.82. PON‐1 = Paraoxonase‐1

Although markers of inflammation and oxidative stress did not differ among the groups (Table 1), PON‐1 activity, a protective antioxidant enzyme, tended to exhibit higher activity levels in the nonuser control group (971.6 ± 169 nmol *p*‐nitrophenol/(min·mL)), intermediate in the e‐cigarette users (682.6 ± 169 nmol *p*‐nitrophenol/(min·mL)), and lowest in the tobacco cigarette users (618.0 ± 125.7 nmol *p*‐nitrophenol/(min·mL)), monotone *P* = 0.10; Fig. 5).

## Discussion

FDG‐PET/CT is a sensitive, noninvasive imaging modality used in many clinical situations, including sarcoidosis, human immunodeficiency virus disease, and fever of unknown origin, to detect the presence of active inflammation (Emami and Tawakol 2014). FDG is taken up by glucose transporters into metabolically active cells such as activated immune cells, including activated macrophages (Tarkin et al. 2014). Blood vessel wall inflammation, characterized by infiltration of activated macrophages, plays a critical role in the initiation and progression of atherosclerosis. Additionally, in preclinical studies, increased sympathetic activity has been shown to activate hematopoietic stems cells in bone marrow, which then replicate as proinflammatory monocytes in the spleen, leaving to infiltrate the blood vessel wall, initiating atherosclerosis (Heidt et al. 2014; Libby et al. 2016). In clinical studies of atherosclerosis using FDG‐PET/CT, increased metabolic activity in hematopoietic tissues, including the bone marrow and spleen, is consistent with activation of the inflammatory Splenocardiac Axis, and has even been shown to confer increased cardiac risk (Tarkin et al. 2014; Emami et al. 2015; Libby et al. 2016). e‐Cigarettes and tobacco cigarettes increase sympathetic activity (Middlekauff et al. 2014; Moheimani et al. 2017), and thus are capable of initiating the Splenocardiac Axis. The major new finding in this study is that FDG uptake is increased in both the spleen and the aorta in a striking linear dose–response relationship from nonsmoking healthy controls to habitual e‐cigarette users to tobacco cigarette smokers. These findings of increased metabolic activity in both spleen and blood vessel wall support the hypothesis of activation of the Splenocardiac Axis in smokers.

Although increased metabolic activity is detectable in the aorta in smokers, the “most diseased segment” (MDS‐TBR) variable was not increased in smokers versus nonsmokers. Perhaps this is not surprising, since the MDS‐TBR analytic approach was developed in patients with known atherosclerosis, which is not likely to be present in our young, otherwise healthy smokers (Tawakol et al. 2013). Rather, in the present study in smokers, we have detected increased metabolic activity in the wall of the aorta, consistent with increased vessel wall inflammation, concerning for the future development of atherosclerosis and cardiac ischemia (Tarkin et al. 2014).

Tobacco cigarette smoking is a risk factor for myocardial ischemia and sudden death, a risk that dissipates shortly – within months to a few years – after quitting (Sandhu et al. 2012; Thun et al. 2013). As supported by this study, activation of the Splenocardiac Axis, which leads to increased numbers of proinflammatory monocytes that infiltrate arterial atheroma, thereby contributing to plaque instability, may underlie this increased risk. It is plausible that the reversal of sympathetic activation that accompanies smoking cessation is also accompanied by a deactivation of the Splenocardiac Axis, explaining the marked fall in cardiac risk following smoking cessation (Harte and Meston 2014). Investigations into hematopoietic tissue and blood vessel metabolic activity in former smokers would be of interest.

From these studies, one cannot be certain which component in tobacco cigarette smoke is the causal agent, but the fact that FDG uptake in hematopoietic tissues was also found to be increased in habitual e‐cigarette users is strongly suggestive of a prominent role for nicotine. Although the carcinogenic toxins present in the aerosol generated by e‐cigarettes are orders of magnitude lower than those present in tobacco smoke (Goniewicz et al. 2014), nicotine levels achieved by each exposure are similar (Goniewicz et al. 2017). In our study, plasma cotinine levels, a metabolite of nicotine with a half‐life of 20 hours and an estimate of nicotine burden, were not different between the tobacco cigarette smokers and e‐cigarette users. Nicotine has a strong sympathomimetic effect, increasing peripheral sympathetic activity, and catecholamine release from the post‐ganglionic nerve terminals and adrenal gland.

Although we did not find biomarker evidence for increased inflammation in our study, Emami and colleagues reported upregulated gene expression of proinflammatory leukocytes and elevated CRP levels in their study of the Splenocardiac Axis (Emami et al. 2015). We did find a trend for decreased PON‐1 activity, a protective antioxidant enzyme that is associated with HDL, in tobacco cigarette and e‐cigarette users compared to controls. This inverse relationship is consistent with our prior report in young women, in whom we found a correlation between decreased PON‐1 activity and increased number of tobacco cigarettes smoked (Ramanathan et al. 2014). Decreased PON‐1 activity has been found to be an independent predictor of premature coronary artery disease in patients younger than 45 years (Sarkar et al. 2006).

### Limitations

Human studies rely on self‐reporting for many of the behaviors that occur outside the laboratory, and thus are vulnerable to inaccuracies (Connor Gorber et al. 2009). To circumvent this, in this study, we required biochemical verification of e‐cigarette use, and absence of tobacco cigarette use (SRNT Subcommittee on Biochemical Verification, 2002; Connor Gorber et al. 2009). Tobacco cigarette exposure is typically quantified by the number of tobacco cigarettes smoked per day, but there is not an equivalent measurement unit for e‐cigarettes. Attempts to quantify e‐cigarette exposure seemed less reliable, since most participants had difficulty quantifying time per day using their e‐cigarette, or milliliters of liquid used per day. The plasma cotinine level measured on the day of the study seemed the most objective means to assess e‐cigarette burden, but remains a rough estimate. Furthermore, some of the e‐cigarette users were former tobacco cigarette users (quit > 1 year). We cannot exclude that certain inflammatory changes were residual from prior tobacco cigarette smoking, persisting for >1 year.

Finally, and importantly, this is a small study. Although the number of FDG‐PET/CT scans performed was not large, it exceeded the number required by our sample size calculations. Furthermore, the FDG‐PET/CT scans were prospectively performed according to a rigorous research protocol designed to maximize count rate and decrease image noise, and were analyzed by a single, expert reader blinded to group affiliation, all ensuring accuracy and consistency. Finally, the finding of a statistically significant, graded increase in FDG activity – lowest in the nonuser, intermediate in the e‐cigarette user, and greatest in the tobacco cigarette smoker, in not only one, but two tissues (spleen and aorta) of the three tissues analyzed makes random variation less likely as the explanation. Nonetheless, as all new research findings warrant, this research warrants replication by other investigators.

## Conclusions

In summary, in this cross‐sectional study of three groups, evidence is presented demonstrating activation of the Splenocardiac Axis in a graded manner from nonuser healthy control subjects to habitual e‐cigarette users to tobacco cigarette smokers. This activation suggests a mechanism by which tobacco cigarettes, and potentially e‐cigarettes, may lead to increased risk of future cardiovascular events.

## Conflict of Interest

None declared.
